# Alterations and interactions: effects of *Porphyromonas gingivalis* and *Bifidobacterium bifidum* on *c-MYC*/*CCAT2* gene expression in colorectal cancer

**DOI:** 10.1186/s13027-026-00786-z

**Published:** 2026-08-11

**Authors:** Kimia Kazemi, Fatemeh Sameni, Amir Abbas Vaezi, Somayeh Yaslianifard, Seidamir Pasha Tabaeian, Reza MohammadSalehi, Hosein Rafiemanesh, Yousef Atefpour, Davood Yadegarynia, Bahareh Hajikhani, Masoud Dadashi

**Affiliations:** 1 Department of Microbiology, School of Medicine, Alborz University of Medical Sciences, Karaj, Iran; 2 Infectious Diseases and Tropical Medicine Research Center, Shahid Beheshti University of Medical Sciences, Tehran, Iran; 3 Department of Internal Medicine, Alborz University of Medical Sciences, Karaj, Iran; 4 Colorectal Research Center, Iran University of Medical Sciences, Tehran, Iran; 5 Molecular Microbiology Research Center, Shahed University, Tehran, Iran; 6 Non-communicable Diseases Research Center, Alborz University of Medical Sciences, Karaj, Iran; 7 Department of Epidemiology and Biostatistics, School of Public Health, Alborz University of Medical Sciences, Karaj, Iran; 8 Department of Microbiology, School of Medicine, Shahid Beheshti University of Medical Sciences, Tehran, Iran

**Keywords:** Colorectal cancer, Long non-coding RNA, *CCAT2*, *c-MYC*, *Porphyromonas gingivalis*, *Bifidobacterium bifidum*

## Abstract

**Background:**

Colorectal cancer (CRC) is one of the most common and life-threatening malignancies worldwide. Despite extensive research, the precise molecular mechanisms underlying CRC progression remain incompletely understood. Accumulating evidence indicates that complex interactions among oncogenic signaling pathways, non-coding RNAs, and the gut microbiota play a pivotal role in CRC pathogenesis. Among oral microbiota, *Porphyromonas gingivalis* (*P. gingivalis*) has been implicated in tumorigenesis through the induction of chronic inflammation and activation of oncogenic pathways. This study aimed to investigate the expression levels of the *lncRNA CCAT2* and the *c-MYC*, as well as to evaluate the relative abundance of *P. gingivalis* and *Bifidobacterium bifidum* (*B. bifidum*) in colorectal cancer tissues compared with adjacent normal tissues.

**Method:**

DNA and RNA were extracted from biopsy specimens obtained from 40 patients with CRC and 25 healthy controls. Complementary DNA (cDNA) was synthesized using commercial kits, and quantitative real-time polymerase chain reaction (qRT-PCR) was conducted to measure the expression levels of *CCAT2* and *c-MYC*, as well as the relative abundance of the 16S rRNA genes of *P. gingivalis* and *B. bifidum*.

**Results:**

Expression levels of *lncRNA CCAT2* and *c-MYC* were significantly higher in CRC tissues than in normal samples (*p* < 0.05). Likewise, the relative abundance of *P. gingivalis* was significantly increased, whereas *B. bifidum* abundance was significantly decreased in CRC tissues (*p* < 0.05). In the combined study population (CRC and healthy controls), Spearman’s rank correlation analysis demonstrated significant positive correlations between *P. gingivalis* abundance and both *CCAT2* and *c-MYC* expression, as well as a significant inverse correlation between *B. bifidum* abundance and *CCAT2* expression. However, in the CRC subgroup, only the positive correlation between *P. gingivalis* abundance and *c-MYC* expression remained statistically significant.

**Conclusion:**

These findings demonstrate significant associations between *P. gingivalis* colonization, *B. bifidum* depletion, and oncogenic molecular alterations in CRC. Although associations involving *CCAT2* were evident in the combined cohort, only the relationship between *P. gingivalis* abundance and *c-MYC* expression persisted within the CRC subgroup, suggesting that some observed correlations may primarily reflect differences between CRC patients and healthy controls. Owing to the cross-sectional design, causal inferences cannot be drawn. Further mechanistic and longitudinal studies are warranted to validate these findings and to explore the clinical utility of microbiota-based biomarkers and therapeutic strategies.

**Graphical Abstract:**

**Figure:**
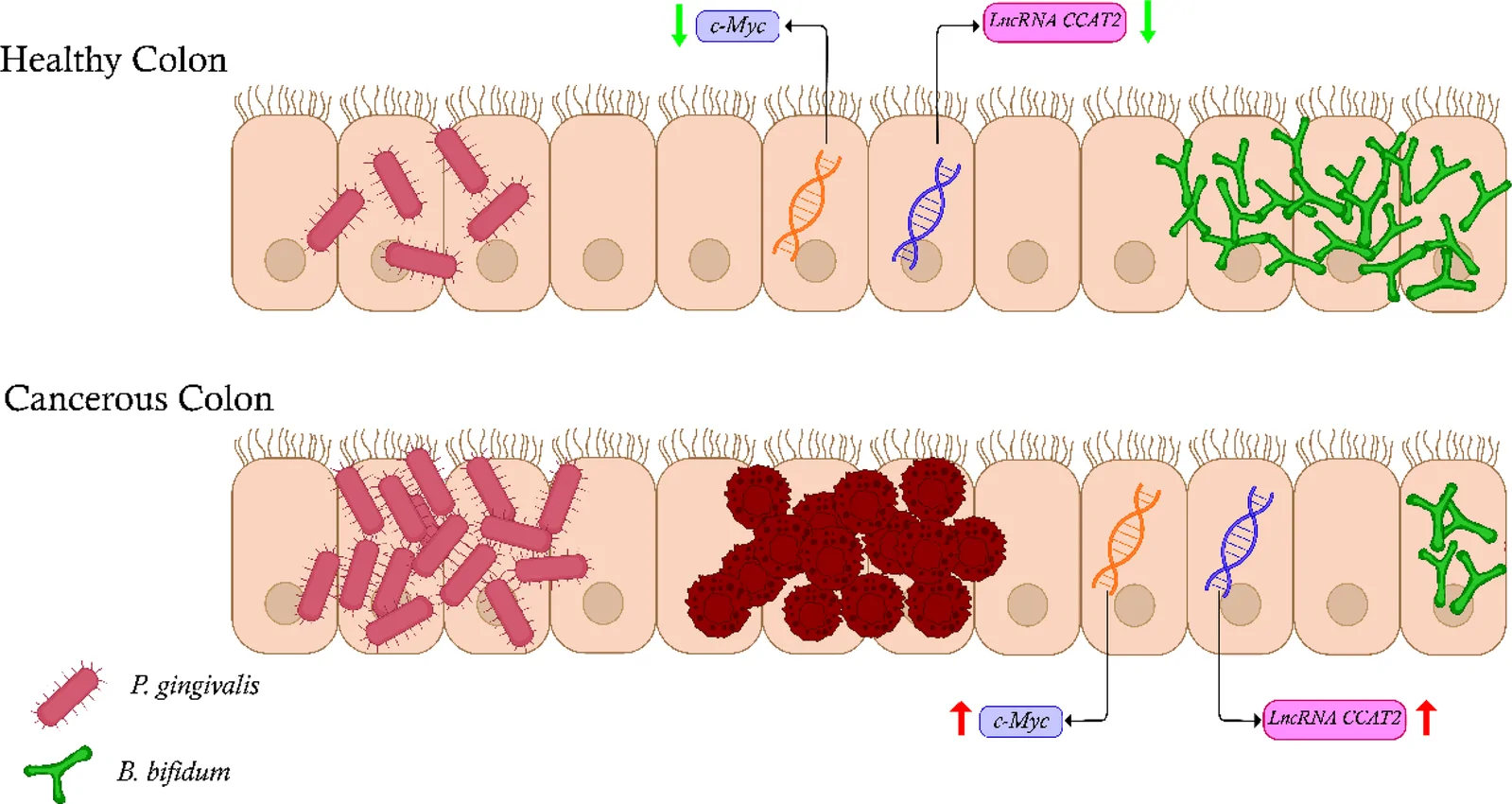

## Introduction

Colorectal cancer is the third most commonly diagnosed malignancy worldwide and represents the second leading cause of cancer-related mortality. Recent global cancer statistics indicate that Europe has the highest age-standardized incidence rates (ASIRs) at 30.4 per 100,000, followed by Oceania (29.8) and North America (26.2), whereas lower incidence rates are observed in Asia (17.6) and Latin America (16.6) [1, 2]. These epidemiological patterns underscore the substantial global burden of CRC. Despite significant advances in epidemiological and molecular research, the precise etiological mechanisms underlying CRC remain incompletely understood. Current evidence suggests that CRC development results from a complex interplay among genetic predisposition, epigenetic alterations, and environmental factors, which collectively disrupt cellular homeostasis and activate oncogenic signaling pathways.

The Wingless/Integrated (Wnt)/β-catenin signaling pathway plays a pivotal role in colorectal carcinogenesis. Under physiological conditions, β-catenin is targeted for degradation by a destruction complex. Upon binding of Wnt ligands to their receptors, this complex is inhibited, leading to β-catenin accumulation and nuclear translocation. In the nucleus, β-catenin activates T-cell factor / lymphoid enhancer factor (TCF/LEF)-dependent transcription of oncogenic target genes, including *c-MYC* and Cyclin D1, thereby promoting tumor cell proliferation, stemness, epithelial–mesenchymal transition (EMT), and resistance to apoptosis [3].

Among the downstream effectors of the Wnt/β-catenin pathway, the cellular myelocytomatosis oncogene (*c-MYC*) plays a central role in tumorigenesis. *c-MYC* encodes a transcription factor that regulates cellular growth, apoptosis, transformation, and therapeutic sensitivity [4, 5]. It is estimated that *c-MYC* controls the transcription of approximately 15% of all human genes, acting as both a transcriptional activator and repressor [6, 7]. *c-MYC* target genes participate in a wide range of cellular functions, including regulation of cell cycle progression, metabolic pathways, protein biosynthesis, cell adhesion, and the expression of non-coding RNAs [6]. Aberrant *c-MYC* expression has been reported in multiple human cancers, including approximately 70–80% of CRC cases [8], and is significantly associated with poor overall survival and adverse prognosis [9–11]. Mechanistically, *c-MYC* promotes aerobic glycolysis by upregulating metabolic genes such as *GLUT1*, *HK2*, *PKM2*, and *LDHA*, thereby supporting rapid tumor growth through the Warburg effect. Recent studies further suggest that indirect inhibition of the β-catenin/*c-MYC* axis, such as enhanced GPC4 degradation via CD36-mediated clearance, can suppress glycolysis and tumor growth, improving clinical outcomes in CRC patients [12]. Disruption of the intestinal microbial community has been increasingly recognized as a significant contributing factor in the development of CRC. Microbial dysbiosis promotes tumorigenesis through impairment of intestinal barrier integrity, induction of chronic inflammation, and activation of oncogenic signaling pathways [13, 14].

Specific microbial species have been implicated in CRC pathogenesis. For instance, *Fusobacterium nucleatum* (*F. nucleatum*) adheres to epithelial cells and activates the Wnt/β-catenin pathway, thereby promoting tumor growth [15–17]. Additionally, *F. nucleatum* modulates the tumor microenvironment by inducing immunosuppression, chronic inflammation, and microbial imbalance, facilitating tumor progression and metastasis [15, 18]. Accumulating evidence further underscores the involvement of the oral microbiota in the progression of CRC, with particular emphasis on *Porphyromonas gingivalis* (*P. gingivalis*). This pathogen promotes tumorigenesis by inducing pro-inflammatory cytokines and disrupting immune signaling, resulting in persistent inflammation and microbial dysbiosis [19, 20].

*P. gingivalis* has been shown to enhance CRC cell proliferation through activation of the mitogen-activated protein kinase (MAPK)/extracellular-signal-regulated kinase (ERK) signaling pathway and upregulation of oncogenic genes, and its increased abundance is associated with poor survival outcomes, suggesting its potential as a negative prognostic biomarker [21].

Mechanistically, *P. gingivalis* activates the NOD-, LRR- and pyrin domain–containing protein 3 (NLRP3) inflammasome, increases Interleukin-1 beta (IL-1β) and caspase-1 expression, alters invariant natural killer T (iNKT) cell function, and recruits immunosuppressive neutrophils, thereby suppressing anti-tumor immunity [19, 22]. Collectively, these findings indicate that *P. gingivalis* contributes to CRC progression through inflammation, immune modulation, and activation of oncogenic pathways.

Conversely, interventions targeting gut microbiota dysbiosis, such as probiotic administration, have shown promise in CRC prevention [23]. Probiotics maintain microbial homeostasis, reinforce the intestinal barrier, modulate immune responses, and produce beneficial metabolites, including short-chain fatty acids (SCFAs), thereby reducing inflammation and carcinogenesis [24–27]. Several studies have demonstrated that probiotics can decrease CRC risk by reshaping the gut microbiome and restoring intestinal balance [28].

*Bifidobacterium* species are well-recognized probiotics associated with gut health and CRC prevention [29]. Composition, increasing anti-inflammatory metabolites such as butyrate, and reducing pro-inflammatory cytokines, including IL-6 and TNF-α [30]. *B. bifidum* has been shown to inhibit tumor growth by suppressing the Wnt/β-catenin pathway and oxidative stress [31–33]. Moreover, stable colonization of *B. bifidum* may prevent precancerous lesions and enhance the efficacy of chemotherapy [34–36].

Long non-coding RNAs (lncRNAs) have been identified as key modulators in the development of CRC, playing significant roles in tumor proliferation, invasion, metastatic spread, and resistance to treatment. Aberrant expression of specific lncRNAs is associated with poor prognosis and increased tumor invasiveness, whereas other lncRNAs function as tumor suppressors whose downregulation activates oncogenic signaling pathways [37, 38]. Among the lncRNAs implicated in colorectal cancer, colon cancer–associated transcript 2 (*CCAT2*) plays a pivotal oncogenic role in tumor progression. *CCAT2* promotes proliferation, migration, invasion, epithelial–mesenchymal transition, and survival of colorectal cancer cells by modulating key signaling pathways, including PI3K/Akt, Wnt/β-catenin, MYC, and GSK3β/β-catenin. Conversely, *CCAT2* knockout or silencing increases apoptosis and suppresses tumor cell growth and metastatic potential, highlighting its value as a promising prognostic biomarker and therapeutic target [39].

Mechanistically, *CCAT2* interacts with TCF7L2 to enhance Wnt/β-catenin signaling, leading to the upregulation of MYC, miR-17-5p, and miR-20a and establishing a positive feedback loop that sustains CRC progression [40].

Clinical studies further support the oncogenic role of *CCAT2*. Elevated serum *CCAT2* levels have been reported in CRC patients compared with healthy controls, with levels decreasing after tumor resection and correlating with tumor burden. Moreover, *CCAT2* is stably detectable in serum exosomes, underscoring its potential as a non‑invasive biomarker for CRC diagnosis and disease monitoring [41]. Collectively, these findings highlight *CCAT2* as a promising molecular target for therapeutic intervention and prognostic assessment in CRC.

Although genetic and microbial factors are increasingly recognized as key contributors to CRC, the interplay between these mechanisms remains poorly understood. A comprehensive characterization of the interactions linking oncogenic signaling pathways, lncRNAs, and the microbiota holds promise for advancing the development of novel diagnostic and therapeutic approaches. Therefore, this study aimed to investigate the association between *CCAT2* and *c-MYC* expression and the abundance of *P. gingivalis* and *B. bifidum* in CRC tissues, with the goal of identifying potential molecular and microbial biomarkers for CRC diagnosis and treatment.

## Materials and methods

### Study design and participants

This case–control study was conducted on biopsy specimens obtained from patients diagnosed with CRC and healthy individuals without any malignancy. The study aimed to compare the expression levels of selected bacterial and host genes between the two groups. The study enrolled patients aged 18 years or older with a histopathologically confirmed diagnosis of CRC. Exclusion criteria comprised the following: (a) lack of written informed consent; (b) prior exposure to systemic chemotherapy or radiotherapy; (c) a concurrent or previous diagnosis of any malignant neoplasm other than CRC.

### Sample collection

Biopsy specimens from colonic and rectal tissues were obtained from CRC patients and healthy individuals by an experienced specialist physician under sterile conditions. Immediately after collection, tissue samples were placed in RNAlater™ Stabilization Solution (Yekta Tajhiz, Iran) to preserve RNA integrity and subsequently stored at − 80 °C until further molecular analyses.

### Genomic DNA extraction

Genomic DNA was isolated from tissue samples using commercial extraction kits (ROJE Technologies, Tehran, Iran) following the manufacturer’s protocol. Nucleic acid concentration and purity were measured using a NanoDrop spectrophotometer (Thermo Fisher Scientific, USA). Purified DNA samples were stored at − 20 °C until further analysis.

### Total RNA extraction and cDNA Synthesis

Extraction of total RNA from tissue samples was carried out with commercial RNA extraction kits (ROJE Technologies, Tehran, Iran) following the manufacturer’s protocols. Following this, first-strand complementary DNA (cDNA) was generated from the isolated RNA by means of a cDNA Synthesis Kit (ROJE Technologies, Tehran, Iran). Each reaction was performed in a final volume of 20 µL, containing 2 µL of RNA template, 2 µL of MixZyme, 10 µL of RT mix, and 6 µL of RT-PCR–grade water.

For cDNA synthesis, the thermal cycling conditions were set as follows: 25 °C for 10 min, 50 °C for 30 min, and a final incubation at 85 °C for 5 min to deactivate the reverse transcriptase enzyme. Synthesized cDNA samples were preserved at − 20 °C until further use.

### Quantitative real-time PCR (qRT-PCR)

Quantitative real-time PCR (qRT-PCR) was performed to assess the expression levels of *P. gingivalis* and *B. bifidum 16S rRNA* genes, as well as *c-MYC* and *lncRNA CCAT2*, in CRC and control samples. Amplification reactions were carried out using a Rotor-Gene Q real-time PCR system (QIAGEN, Germany).

Each 20 µL reaction mixture contained 10 µL of 2× SYBR Green Master Mix (ROJE Technologies, Tehran, Iran), 1 µM of each forward and reverse primer, 2 µL of cDNA template, and RNase-free water to reach the final volume. Primer sequences are listed in Table 1, and the optimized thermal cycling conditions are provided in Table 2.

All reactions were performed in duplicate for both patient and control samples to ensure reproducibility. Amplification specificity was confirmed by melting-curve analysis, which demonstrated a single specific peak for positive reactions. Samples with Ct values < 35 and a specific melting profile were considered positive. The universal bacterial 16S rRNA gene and glyceraldehyde-3-phosphate dehydrogenase (GAPDH) were used as internal reference genes for normalization.

### Questionnaire and data collection

A structured, researcher-designed questionnaire was administered to all participants to collect demographic and baseline clinical data. Additional information relevant to the study objectives, including probiotic consumption history and previous oral and dental diseases, was also obtained. Questionnaires were self-administered under researcher supervision to ensure completeness and accuracy. Participation was voluntary, and all collected data were anonymized and used exclusively for research purposes in accordance with ethical approval from the institutional review board.

### Data analysis

Relative gene expression levels were calculated using the comparative 2⁻ΔΔCt method, as follows:

$$\eqalign{& {\rm{Relative }}\,{\rm{Expression}} \cr & \,\,\, = {{{2^ - }\left( {CT\,target - CT\,reference} \right)Tumor} \over {{2^ - }\left( {CT\,target - CT\,reference} \right)Normal}} \cr} $$

The statistical analysis of the data was performed using SPSS version 21.0 (IBM Corp., Armonk, NY, USA) and GraphPad Prism version 8.0 (GraphPad Software, San Diego, CA, USA). The normality of the distribution of continuous variables was tested with the Kolmogorov-Smirnov and Shapiro-Wilk tests. Based on the normality results, comparisons between two groups were made using either the independent-samples t-test (for parametric data) or the Mann–Whitney U test (for non-parametric data).

Statistical analyses comparing gene expression levels between groups were performed using ΔCt values. The 2^^−ΔΔCt^ method was applied solely for the calculation and graphical representation of relative fold-changes in gene expression.

Given the targeted hypothesis-driven nature of the study and the limited number of predefined outcomes, p-values were interpreted at a nominal significance level (*p* < 0.05) without adjustment for multiple comparisons.

A multivariable binary logistic regression model was used to assess the independent association between microbial markers and CRC. CRC status (case/control) was included as the dependent variable, while age, sex, *P. gingivalis* abundance, and *B. bifidum* abundance were entered as independent variables. Adjusted odds ratios (aORs) and 95% confidence intervals (CIs) were calculated. Due to missing demographic and molecular data, a complete-case analysis was performed, and only participants with complete information were included in the model.

Spearman correlation analysis was performed to assess the relationship between *P. gingivalis* abundance and gene expression levels (*c-MYC* and *lncRNA CCAT2*). Correlation coefficients (r) and two-tailed p-values were calculated.

## Results

### Study samples and demographic characteristics

**Fig. 1:**
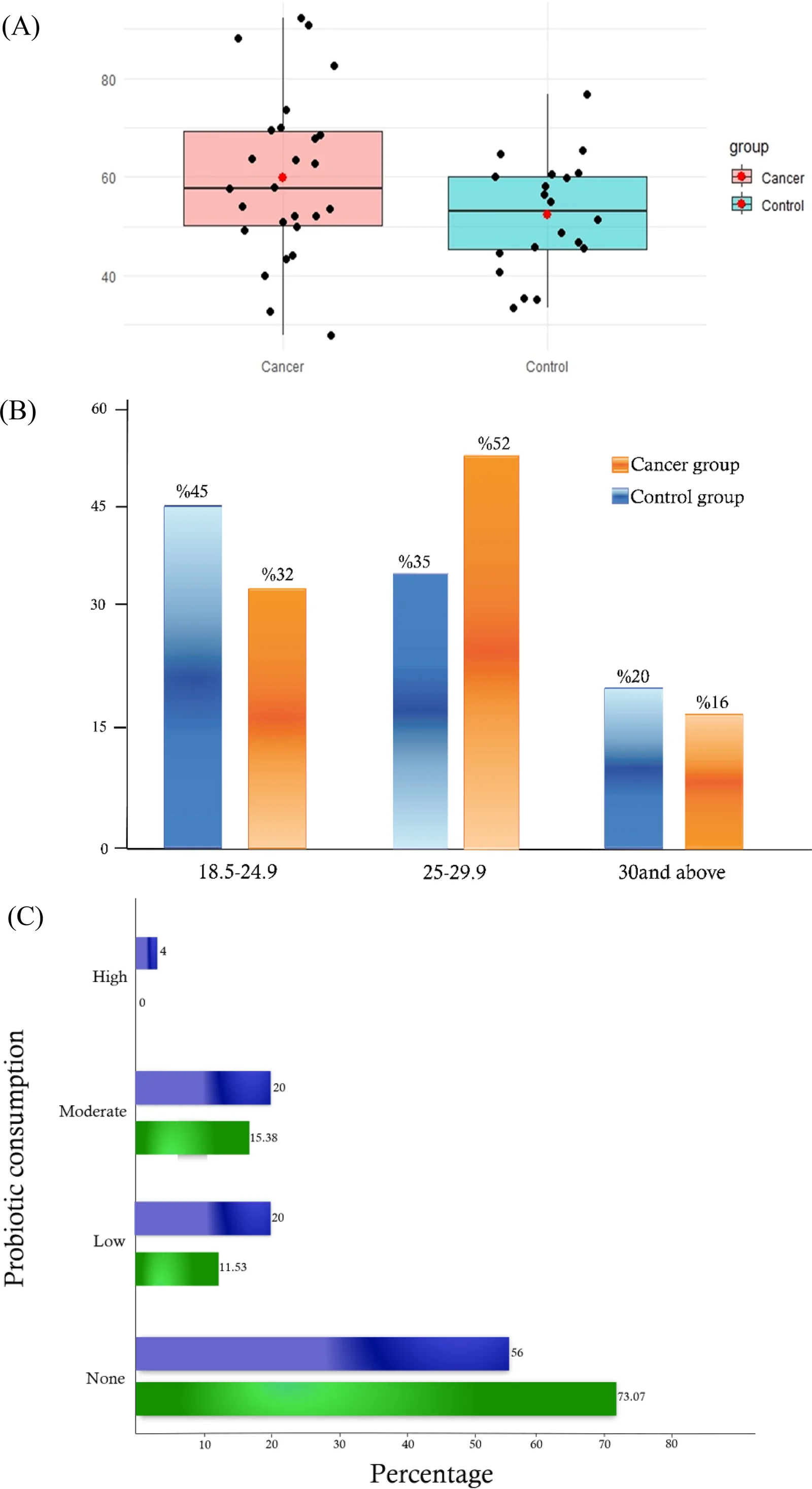
Age (**A**) and BMI (**B**) distributions, and probiotic consumption rate (**C**) in the study groups

In this study, a total of 40 colorectal tissue samples from patients with colorectal cancer (CRC) and 25 samples from healthy individuals were included as the control group. Among CRC patients, 62.16% were male and 37.83% were female. The distribution of gender did not differ significantly between the case and control groups (*P* = 0.07). The mean age of the CRC patients was 61.6 ± 17.1 years, whereas the mean age of the control group was 49.5 ± 12.6 years. A significant age difference was observed between the two groups, with CRC patients being older than controls (*P* = 0.008). Figure 1A illustrates the age distribution of the study participants, while Fig. 1B depicts the Body Mass Index (BMI) distribution in both groups.

### Questionnaire data

The results of the questionnaire-based assessments are summarized in Table 3. A history of gastrointestinal disease was reported in 32.14% of CRC patients and 76% of healthy controls, revealing a statistically significant difference between the two groups (*P* = 0.001). In the CRC group, peptic ulcer disease was the most prevalent gastrointestinal disorder (14.28%). In contrast, irritable bowel syndrome and colitis were the most commonly reported conditions among controls, each with a prevalence of 12%. Detailed information regarding the types and prevalence of gastrointestinal diseases is provided in Table 4.

Overall, 57.14% of CRC patients and 36% of controls reported at least one underlying disease; however, this difference was not statistically significant (*P* = 0.17). The prevalence and types of underlying conditions are presented in Table 5.

A history of probiotic consumption was reported by 26.92% of CRC patients and 40% of individuals in the control group. No statistically significant differences were observed between the groups at any level of probiotic intake. The distribution of probiotic consumption in the two groups is illustrated in Fig. 1C.

### Location of cancerous masses and their histopathological types

**Fig. 2:**
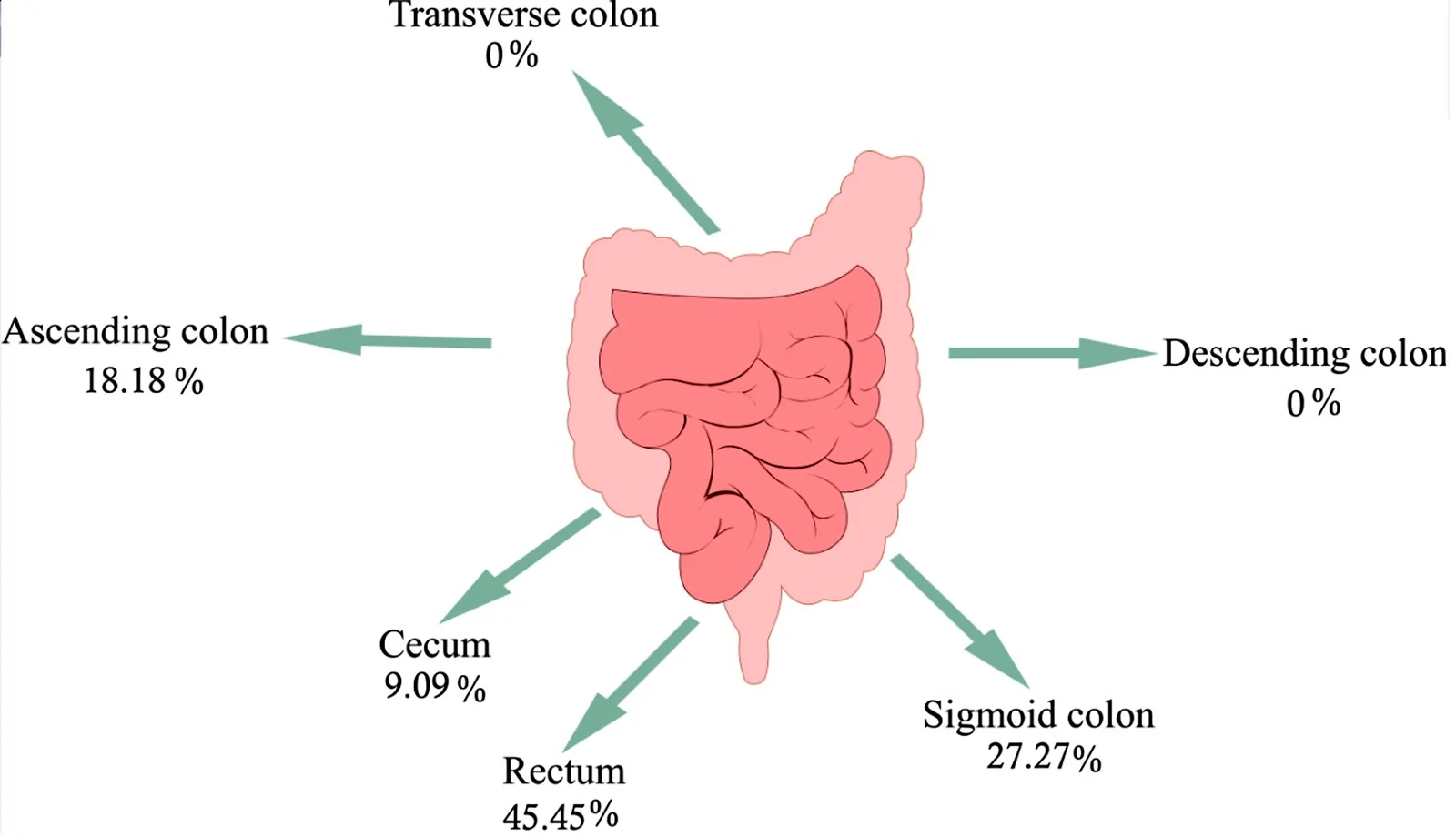
Distribution of cancerous lesions in different regions of the colon and rectum

The majority of cancerous lesions were located in the rectum (45.45%), followed by the sigmoid colon (27.27%), ascending colon (18.18%), and cecum (9.09%). Histopathological examination revealed that 77.27% of tumors were adenocarcinomas, whereas 22.72% were adenomas. The anatomical distribution of tumors is shown in Fig. 2.

### Relative expression of *c-MYC*

The expression levels of the *c-MYC* gene were significantly higher in CRC tissues than in normal tissues, as determined by quantitative real‑time PCR (*P* = 0.0002).

**Fig. 3:**
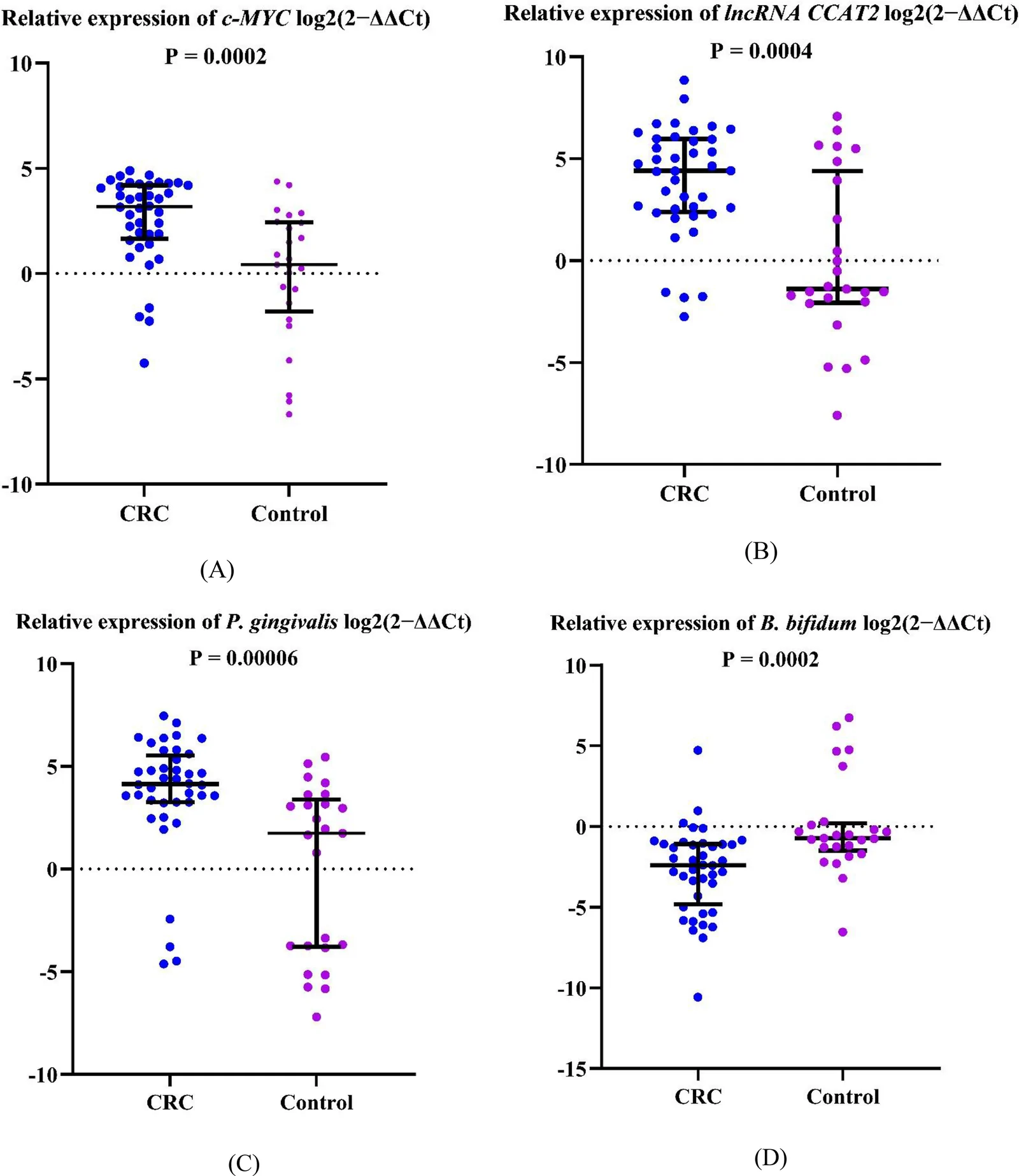
Comparison of *c-MYC* (**A**), *lncRNA CCAT2* (**B**), *P. gingivalis* 16S rRNA (**C**), and *B. bifidum* 16S rRNA (**D**) expression levels between patients and controls

 The comparative expression levels of *c-MYC* between patients and controls are shown in Fig. 3A.

### Relative expression of *lncRNA CCAT2*

Similarly, *lncRNA CCAT2* expression was significantly elevated in CRC tissues compared with healthy controls (*P* = 0.0004). The comparative expression levels of *lncRNA CCAT2* are depicted in Fig. 3B.

### Fold change analysis of *c-MYC* and *lncRNA CCAT2* genes

**Fig. 4:**
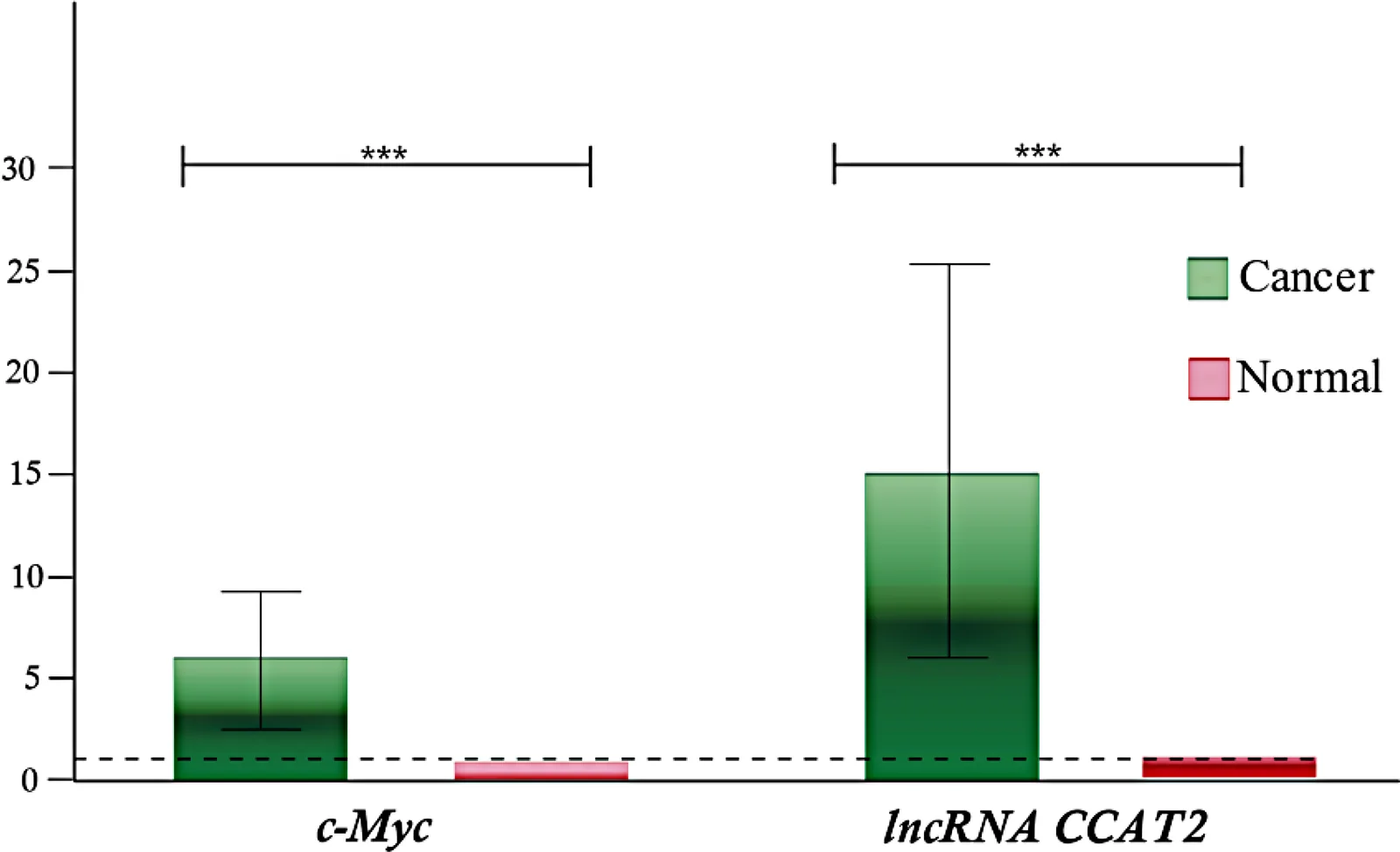
Fold changes of *c-MYC* and *lncRNA CCAT2* expression levels in patient and control groups

Fold change analysis revealed that *c-MYC* expression was increased by 5.91-fold in CRC tissues relative to normal samples (*P* = 0.0008). Likewise, *lncRNA CCAT2* expression exhibited a 15-fold increase in CRC tissues (*P* = 0.0001), as illustrated in Fig. 4.

### The relative expression level of the *P. gingivalis* 16S rRNA gene

The relative abundance of *P. gingivalis* was assessed using a quantitative real-time PCR assay targeting the species-specific 16S rRNA gene. CRC tissues exhibited significantly higher *P. gingivalis* levels compared with normal tissues, with median values of 0.007 (0.004–0.01) and 0.001 (0.0003–0.004), respectively (*P* = 0.00006, Mann–Whitney test; Fig. 3C). Receiver Operating Characteristic (ROC) curve analysis was used to determine the optimal cutoff value for *P. gingivalis* abundance, allowing classification of samples into low- and high-level groups. Among the 36 CRC samples positive for *P. gingivalis*, 32 were categorized as high-level and 4 as low-level.

Using the ROC-derived cutoff, high *P. gingivalis* expression was observed in 88.9% of CRC tissues compared with 40% of control samples.

Within the CRC group, the mean age of patients with high *P. gingivalis* levels was 60.57 ± 15.08 years, which was 1.9 years lower than that of patients with low levels (62.5 ± 21.92 years). In the high-level group, 64.28% were male and 35.71% were female, with no significant difference in gender distribution between high- and low-level groups (*P* > 0.05).

### Frequency of *P. gingivalis* in patient and control groups

*P. gingivalis* was detected in 90% of CRC samples and 60% of control samples. This difference was statistically significant (Chi-square test, *P* = 0.006), with an odds ratio (OR) indicating that CRC patients were approximately six times more likely to harbor *P. gingivalis* than controls (OR = 6.0; 95% CI: 1.62–22.16).

**Fig. 5:**
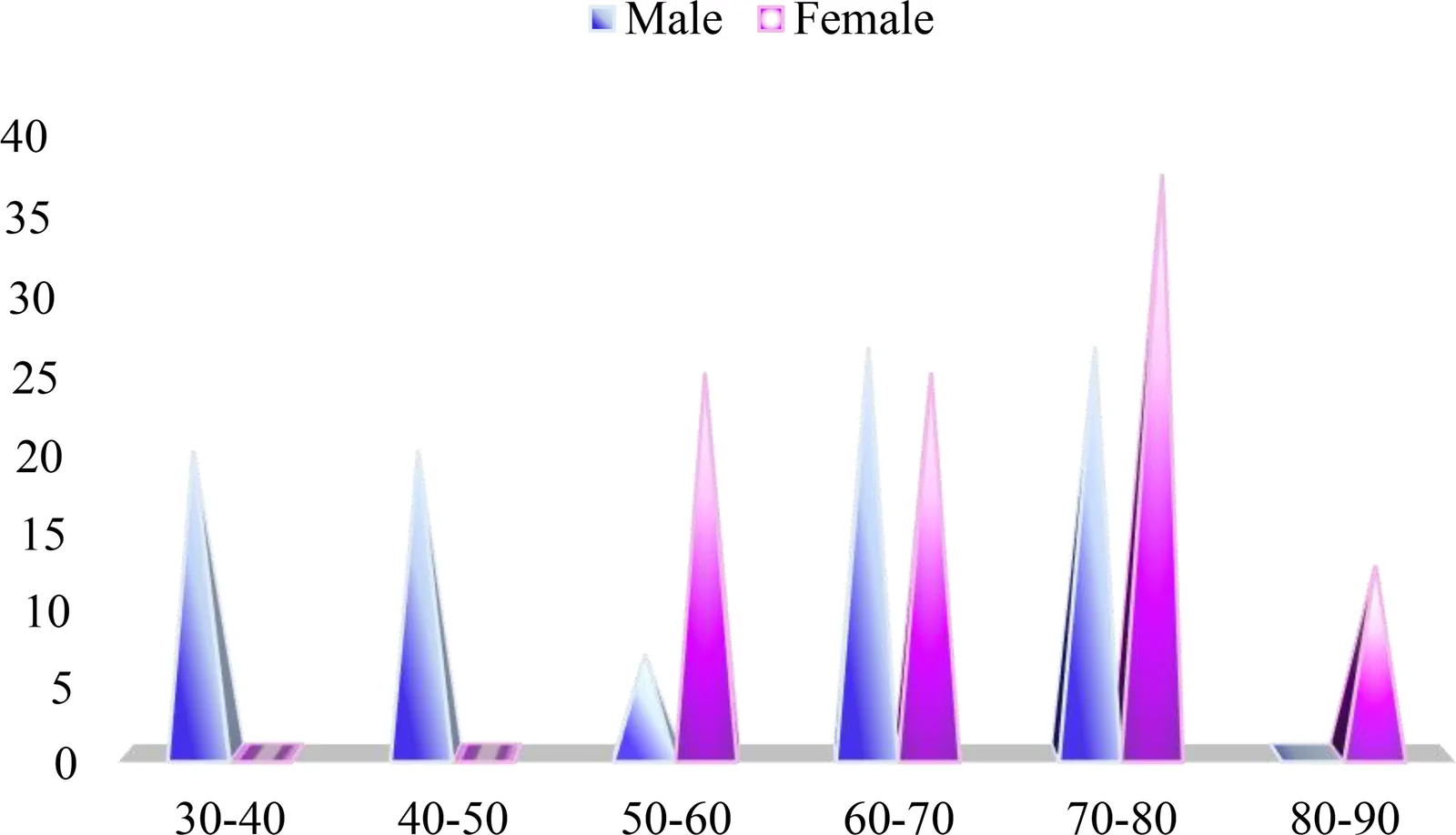
Prevalence of *P. gingivalis* across age groups stratified by gender

Among *P. gingivalis*–positive CRC samples, 59.37% were obtained from male patients and 40.62% from female patients. No significant difference in *P. gingivalis* prevalence was observed between female CRC patients and female controls (*P* = 0.68). However, a significant difference was detected among males (*P* = 0.008). The age distribution of *P. gingivalis*–positive patients is shown in Fig. 5.

### Expression of *c-MYC* and *lncRNA CCAT2* in *P. gingivalis-*Positive and -Negative Samples

**Fig. 6:**
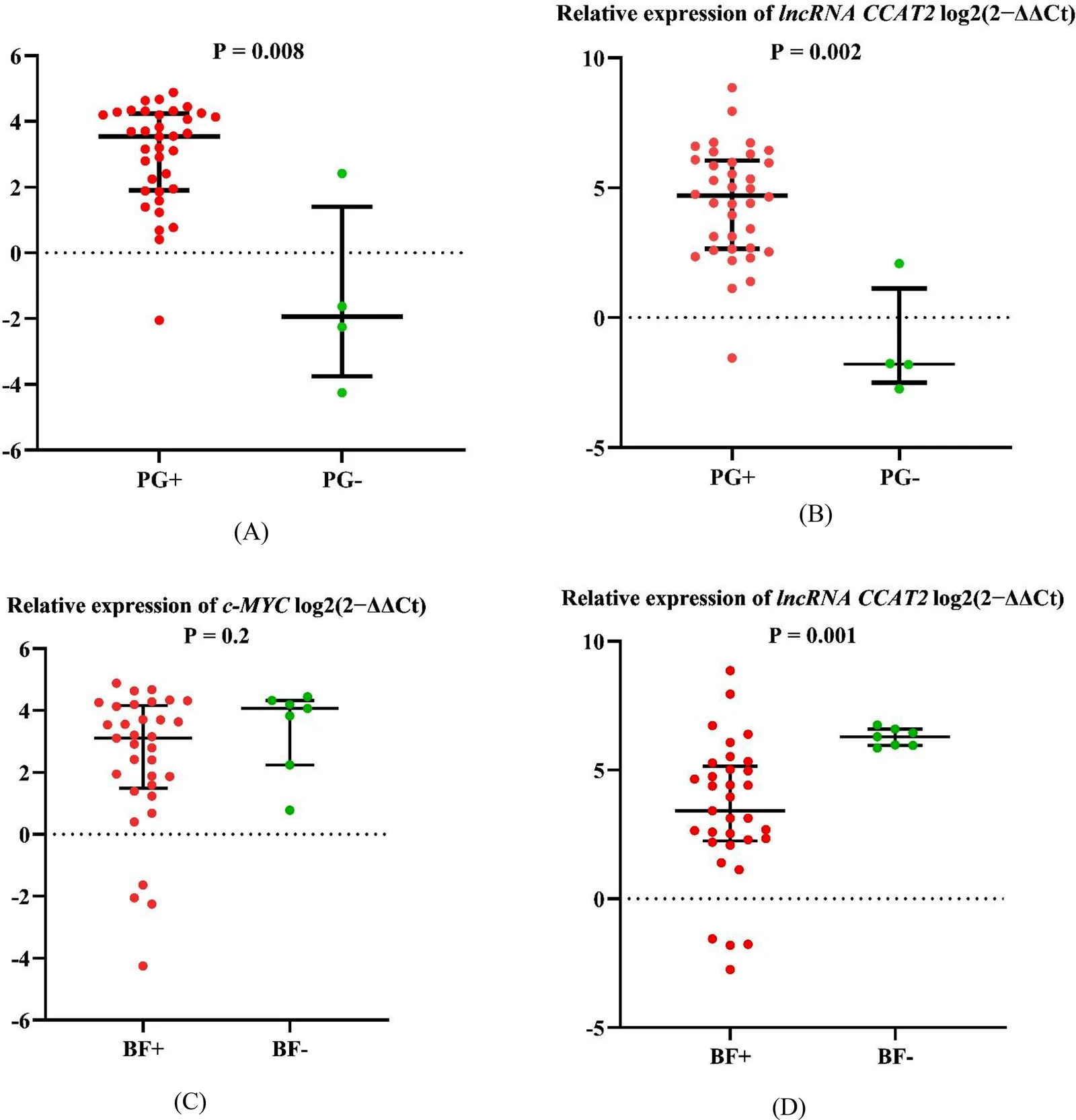
Relative *c-MYC* (**A**) and *lncRNA CCAT2* (**B**) expression in *P. gingivalis*–positive and –negative cancer samples. Relative *c-MYC* (**C**) and *lncRNA CCAT2* (**D**) expression in *B. bifidum*–positive and –negative cancer samples

CRC samples positive for *P. gingivalis* exhibited significantly higher *c-MYC* expression compared with negative samples (*P* = 0.008; Fig. 6A). Similarly, *lncRNA CCAT2* expression was markedly elevated in *P. gingivalis*–positive tissues, with a highly significant difference between the two groups (*P* = 0.002; Fig. 6B).

### Relative expression level of the *B. bifidum* 16S rRNA gene

The relative abundance of *B. bifidum* was evaluated using quantitative real-time PCR. Normal tissues exhibited significantly higher *B. bifidum* levels compared with CRC tissues, with median values of 0.0008 (0.0005–0.001) and 0.0002 (0.00004–0.0006), respectively (*P* = 0.0002, Mann–Whitney test; Fig. 3D).

ROC curve analysis was used to classify samples into low- and high-abundance groups. Among 33 CRC samples positive for *B. bifidum*, 5 were categorized as high-level and 28 as low-level. High *B. bifidum* abundance was significantly less frequent in CRC tissues than in normal controls (15.2% vs. 62.5%).

### Frequency of *B. bifidum* in patient and control groups

*B. bifidum* was detected in 82.5% of CRC samples and 96% of control samples; however, this difference was not statistically significant (Fisher’s exact test, *P* = 0.14). CRC patients were approximately five times more likely to be negative for *B. bifidum* than controls (OR = 5.09; 95% CI: 0.59–44.16), though this finding should be interpreted with caution due to the limited sample size.

**Fig. 7:**
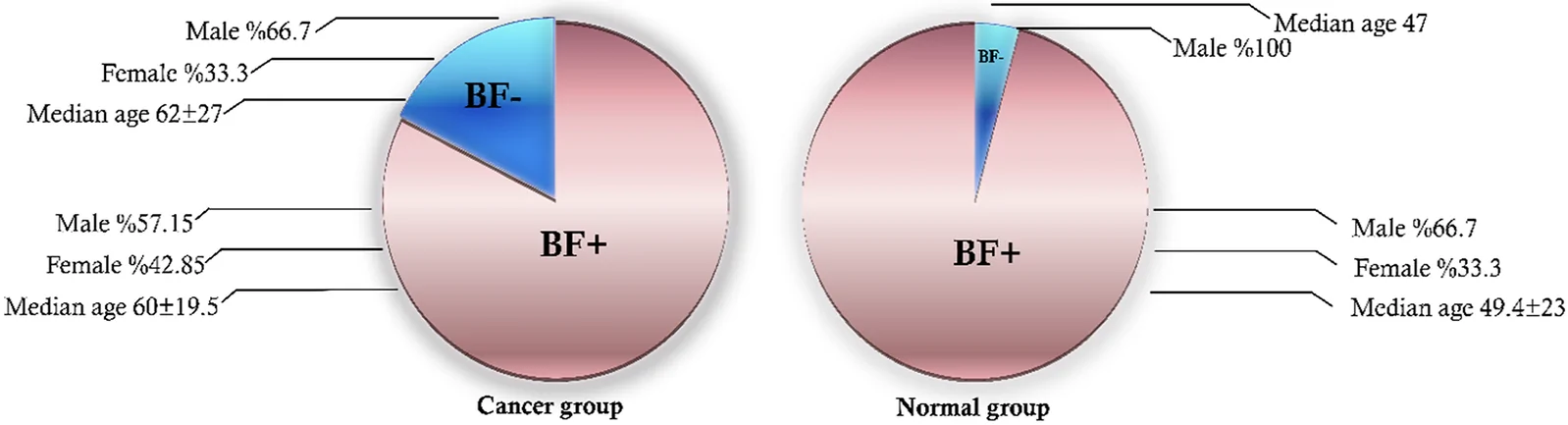
Distribution of *B. bifidum* presence in cancer and normal groups. Pie charts show the proportion of *B. bifidum* positive (BF+) and *B. bifidum* negative (BF-) individuals in each group, along with sex (%), and median age

Among *B. bifidum*–positive CRC samples, 57.14% were from male patients and 42.85% from female patients. In the control group, 33.3% of *B. bifidum*–positive samples were from males and 66.6% from females. Age distributions are shown in Fig. 7. No significant differences in *B. bifidum* prevalence were observed between CRC and control groups when stratified by sex (*P* > 0.05).

### Relative expression of *c-MYC* and *lncRNA CCAT2* in *B. bifidum*–Positive and –Negative Samples

Analysis of *c-MYC* expression revealed higher levels in *B. bifidum*–negative CRC samples compared with positive samples; however, this difference was not statistically significant (*P* = 0.2; Fig. 6C). In contrast, *lncRNA CCAT2* expression was significantly lower in *B. bifidum*–positive samples than in negative samples (*P* = 0.001; Fig. 6D).

### Multivariable logistic regression analysis

Age was independently associated with CRC (aOR = 1.091, 95% CI: 1.012–1.176, *p* = 0.023), whereas sex was not significantly associated with CRC (aOR = 2.278, 95% CI: 0.316–16.405, *p* = 0.414).

Among the microbial markers, *P. gingivalis* was positively associated with CRC (aOR = 13.860, 95% CI: 1.926–99.756, *p* = 0.009), while *B. bifidum* showed an inverse association (aOR = 0.135, 95% CI: 0.021–0.866, *p* = 0.035). These associations remained significant after adjustment for age and sex.

### Correlation analysis

Spearman’s rank correlation analysis was performed to assess the relationships among *c-MYC* expression, *lncRNA CCAT2* expression, *P. gingivalis* abundance, and *B. bifidum* expression in the combined study population, including both CRC patients and healthy controls (*n* = 65).

**Fig. 8:**
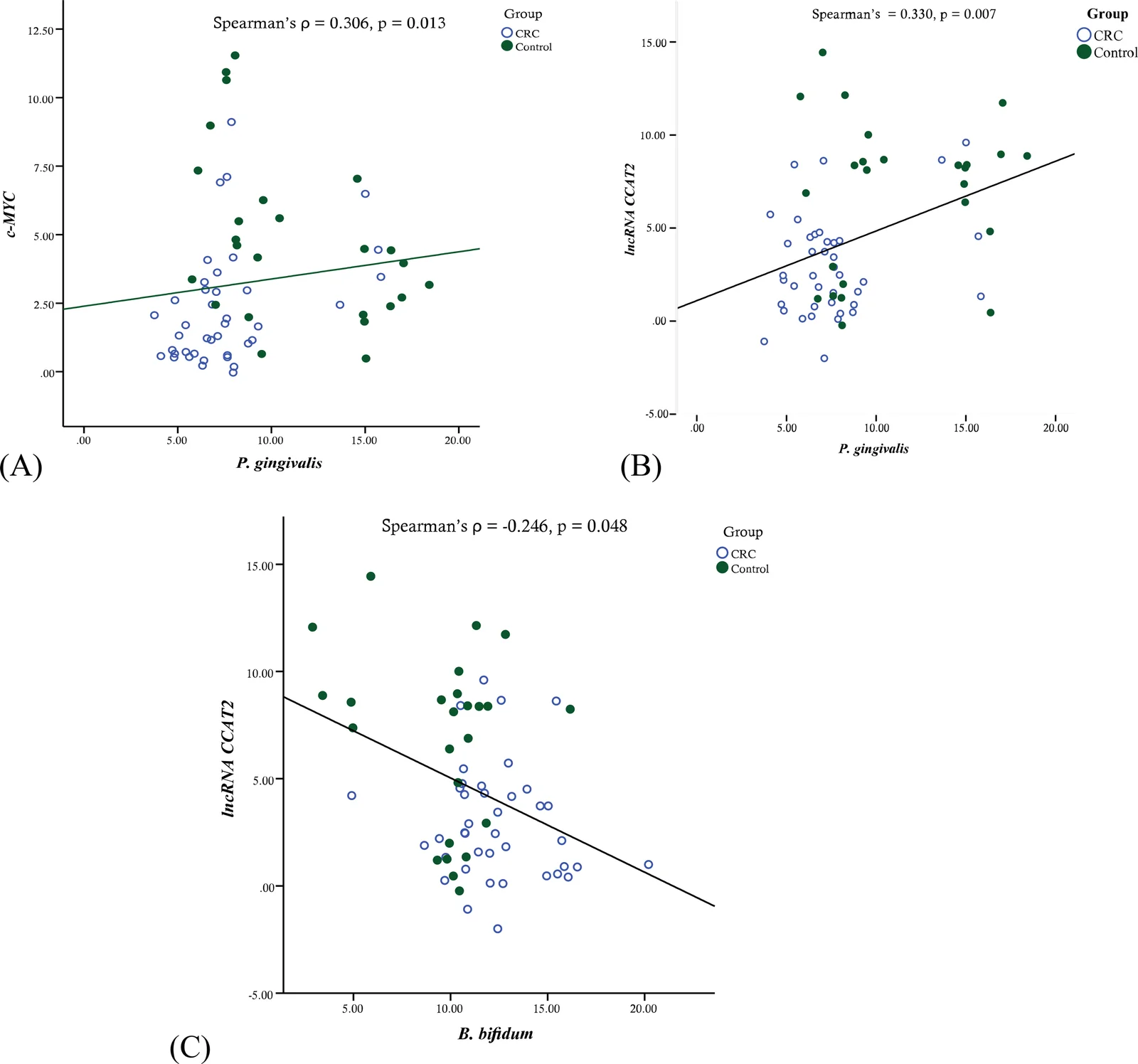
Correlation analysis of *P. gingivalis* and *c-MYC* (**A**), *lncRNA CCAT2* (**B**), and *B. bifidum* and *lncRNA CCAT2* (**C**) expression levels in CRC and control subjects

In patients and healthy controls, significant positive correlations were observed between *P. gingivalis* abundance and both *c-MYC* expression (rₛ = 0.306, *p* = 0.013) and *lncRNA CCAT2* expression (rₛ = 0.330, *p* = 0.007). In addition, a significant negative correlation was found between *lncRNA CCAT2* expression and *B. bifidum* expression (rₛ = −0.246, *p* = 0.048) (Fig. 8). No significant correlations were observed among the remaining variables.

Subgroup analysis was then performed separately in CRC patients (*n* = 40). A significant positive correlation was identified between *c-MYC* expression and *P. gingivalis* abundance (rₛ = 0.332, *p* = 0.036), whereas no other significant correlations were detected.

Overall, the association between *P. gingivalis* abundance and *c-MYC* expression was consistently observed in both the combined cohort and the CRC subgroup, while associations involving *lncRNA CCAT2* and *B. bifidum* were not evident within the CRC group alone.

## Discussion

CRC is the second most common cause of cancer-related death globally [2]. Despite its high prevalence and fatality rate, many of the molecular pathways and genetic alterations involved in CRC initiation and progression remain incompletely understood. CRC develops through a complex interplay of genetic susceptibility, environmental exposures, and alterations in the intestinal microbiota. Non-modifiable risk factors, including age, sex, genetic predisposition, and family history, play a substantial role in CRC risk, while modifiable factors such as diet, obesity, smoking, and alcohol consumption also contribute to disease development [2]. Recent evidence highlights the dual role of the gut microbiota in CRC pathogenesis: pathogenic bacteria may promote tumorigenesis by activating oncogenic signaling pathways [42], whereas probiotic and commensal species may exert protective effects by modulating inflammation, preserving epithelial barrier integrity, and regulating host gene expression [43]. In this context, the present study investigated the association between the abundance of *Porphyromonas gingivalis* and *Bifidobacterium bifidum* and the expression of the oncogenes *c-MYC* and *lncRNA CCAT2* in CRC tissues.

In the present study, we evaluated the relationship between cigarette smoking, alcohol consumption, and CRC risk. Our findings showed no statistically significant differences in smoking patterns or alcohol consumption between CRC patients and healthy controls. These results are consistent with previous studies reporting that overall alcohol intake does not significantly differentiate CRC molecular subtypes and that, although smoking may be associated with specific molecular pathways, its overall impact on CRC risk remains inconsistent [44].

The lack of significant associations observed in our study may be explained by the relatively small sample size, the low prevalence of high-risk consumption patterns in the study population, or population-specific differences in tumor biology. Therefore, larger studies incorporating molecular stratification are warranted to clarify the contribution of smoking and alcohol consumption to CRC development.

We also assessed probiotic consumption patterns in CRC patients and healthy controls using questionnaire data. Although differences in probiotic consumption were observed between the groups-specifically, higher rates of non-consumption among CRC patients and high-level consumption exclusively among controls-no statistically significant differences were detected at any consumption level. These findings partially contrast with epidemiological studies reporting an inverse association between yogurt intake and CRC risk [45]. While several studies suggest that probiotics may provide clinical benefits during postoperative CRC management, their role in preventing CRC onset remains uncertain [46]. Notably, our molecular findings demonstrated significantly higher *B. bifidum* abundance in healthy individuals compared with CRC patients, suggesting that gut microbiota composition may be more critical than probiotic intake alone. Future studies should therefore evaluate both probiotic consumption and microbiota structure to achieve a more comprehensive understanding of the role of probiotics in CRC prevention.

This study further investigated the abundance of *P. gingivalis* in CRC and normal tissues and its association with *c-MYC* and *lncRNA CCAT2* expression. Previous experimental studies have demonstrated that *P. gingivalis* infection activates the MAPK/ERK signaling pathway through upregulation of key components such as KRAS, BRAF, MEK, ERK, and c-Fos, thereby promoting CRC cell proliferation and survival [21].

These findings highlight the importance of MAPK/ERK signaling in CRC pathogenesis and suggest that *P. gingivalis* may facilitate tumor progression by activating oncogenic pathways.

Clinical evidence also supports the involvement of *P. gingivalis* in CRC. Kerdreux et al. reported a prevalence of 2.6–5.3% among CRC patients, with significantly higher levels detected in fecal samples compared with healthy individuals. Importantly, *P. gingivalis* colonization was associated with the microsatellite instability (MSI) subtype and shorter cancer-specific survival [47].

In contrast, the prevalence of *P. gingivalis* observed in the present study was approximately 90%, which is considerably higher than previously reported. This discrepancy may be attributed to differences in sample type, as our study analyzed intestinal biopsy specimens rather than fecal samples, potentially providing a more accurate representation of mucosa-associated bacterial populations. Additionally, geographic, ethnic, and lifestyle differences may contribute to variability in *P. gingivalis* prevalence. To our knowledge, this is the first study to assess *P. gingivalis* abundance in CRC patients from Iran, limiting direct regional comparisons.

Consistent with earlier reports, we observed significantly higher *P. gingivalis* abundance in CRC tissues compared with normal controls, and elevated bacterial levels were associated with a markedly increased risk of CRC. While previous studies primarily focused on *P. gingivalis*–mediated activation of inflammatory pathways, including the NLRP3 inflammasome [19], our findings extend this evidence by demonstrating significantly higher expression of *c-MYC* and *lncRNA CCAT2* in *P. gingivalis*–positive tumors. These results suggest that *P. gingivalis* may contribute to CRC progression not only through inflammation but also by modulating oncogenic gene expression.

Conversely, our data revealed a significant reduction of *B. bifidum* in CRC tissues, with low abundance strongly associated with increased CRC risk. These findings align with in vitro studies showing that *Bifidobacterium* species exert anticancer effects by inducing apoptosis and regulating pro- and anti-apoptotic genes [31]. Moreover, the observed inverse relationship between *B. bifidum* abundance and *CCAT2* expression suggests a potential mechanistic link between beneficial microbiota and suppression of oncogenic pathways.

Supporting this hypothesis, Heydari et al. demonstrated that oral administration of *B. bifidum* in a mouse model of colon cancer modulated tumor-related molecular regulators, including downregulation of oncogenic miRNAs and KRAS expression, indicating a protective effect against colon carcinogenesis [48]. Our clinical findings complement these experimental data by showing that *B. bifidum* depletion in human CRC tissues is associated with elevated oncogenic markers, particularly *lncRNA CCAT2*.

Although *B. bifidum* was detected in a high proportion of both CRC and normal samples, its overall abundance was significantly lower in CRC tissues. This observation is consistent with previous reports of reduced *Bifidobacterium* levels in CRC-associated dysbiosis [49]. Collectively, these findings suggest that *B. bifidum* may protect against colorectal tumorigenesis through multiple mechanisms, including modulation of non-coding RNAs and suppression of oncogenic signaling.

We also observed a nearly 15-fold upregulation of *lncRNA CCAT2* in CRC tissues compared with healthy controls. Although similar overexpression has been reported previously in Iranian populations [50], the magnitude observed in our study was substantially higher. This difference may be attributed to methodological variations, particularly the use of healthy tissues rather than adjacent non-tumorous tissues as controls, which may already be influenced by the tumor microenvironment. Functional studies have demonstrated that *CCAT2* promotes CRC cell proliferation and inhibits apoptosis [51], supporting its oncogenic role and reinforcing its potential clinical relevance.

Taken together, these findings underscore the significance of *lncRNA CCAT2* as a potential diagnostic and therapeutic biomarker in CRC. The strong association between *CCAT2* expression, pathogenic microbiota enrichment, and tumor presence suggests a coordinated molecular–microbial axis contributing to CRC pathogenesis.

Correlation analysis revealed significant positive associations between *P. gingivalis* abundance and both *c-MYC* and *CCAT2* expression, while an inverse association was observed between *CCAT2* expression and *B. bifidum*. These findings suggest that microbial composition may be linked to host gene expression patterns related to proliferation and tumor-associated regulatory pathways. Importantly, this pooled analysis was performed to explore overall system-level associations across the study population rather than to infer disease-specific effects.

To account for potential confounding introduced by disease status, a separate analysis was conducted in CRC patients. In this subgroup, only the positive association between *P. gingivalis* abundance and *c-MYC* expression remained statistically significant, whereas all other correlations lost significance. This suggests that the relationship between *P. gingivalis* and *c-MYC* may represent a more robust and disease-relevant interaction, persisting even within the tumor context.

The disappearance of the associations involving *CCAT2* and *B. bifidum* in the CRC subgroup may reflect the extensive molecular and microbial dysregulation characteristic of colorectal carcinogenesis. In cancerous tissue, regulatory networks are often altered by genetic, epigenetic, and tumor microenvironment–driven mechanisms, which may override or obscure baseline microbiota–host gene expression relationships observed in the combined cohort.

Taken together, the results indicate that while microbiota–host gene associations can be detected at the system level in the overall population, only the relationship between *P. gingivalis* and *c-MYC* appears to be consistently maintained in CRC patients. This supports a potential role for *P. gingivalis* in modulating oncogenic signaling pathways, particularly those involving *c-MYC*, in colorectal carcinogenesis.

Future studies with larger cohorts and functional experiments are required to clarify the causal nature of these associations and to determine whether microbial modulation of host gene expression contributes directly to CRC development.

### Limitations

This study has several limitations. First, long-term clinical follow-up was not feasible, precluding assessment of patient outcomes, survival, and treatment response. Second, although major confounding factors such as age, sex, and BMI were considered, residual confounding from unmeasured variables, including dietary habits, medication use, and other lifestyle factors, cannot be excluded. Third, the use of targeted PCR-based assays rather than high-throughput sequencing approaches limited comprehensive characterization of the gut microbiota and its functional potential.

In addition, detailed clinicopathological parameters, including TNM stage, histological grade, and microsatellite instability (MSI) status, were not available because samples were obtained at the biopsy stage prior to comprehensive pathological characterization. The exact anatomical location of biopsy collection was also unavailable for a proportion of healthy control samples, preventing site-matched comparisons between control and CRC tissues. Given the known regional heterogeneity of the colorectal microbiota, this may have introduced additional variability into the microbial analyses.

Furthermore, due to missing demographic and molecular data, multivariable analyses were performed using complete-case data, resulting in a reduced sample size for adjusted analyses.

Additionally, the relatively modest sample size and the absence of an a priori power calculation may have limited the statistical power of subgroup, correlation, and multivariable analyses. As reflected by the wide confidence intervals observed for some odds ratio estimates, the precision of the estimated effect sizes was limited. Therefore, the magnitude of the reported associations should be interpreted cautiously until validated in larger, adequately powered cohorts.

Finally, no formal adjustment for multiple comparisons was applied; therefore, the possibility of type I error should be considered when interpreting the findings. Future studies with larger cohorts, comprehensive clinicopathological data, site-matched sampling, and longitudinal follow-up are warranted to validate and extend the present observations.

## Conclusion

This study demonstrates that oral and gut microbiota, particularly *P. gingivalis* was significantly associated with colorectal cancer and with increased expression of *c-MYC* and *lncRNA CCAT2*. In contrast, *B. bifidum* was more abundant in healthy individuals, suggesting a potential protective role against CRC. The marked upregulation of *lncRNA CCAT2* in CRC tissues highlights its promise as a non-invasive biomarker for early detection and disease monitoring. Collectively, these findings emphasize the importance of microbial–molecular interactions in CRC pathogenesis and support further investigation into microbiota-based diagnostic and therapeutic strategies for CRC prevention and treatment.

**Table 1:** Primers used in this study

Gene		Sequence	Reference
*16 S rRNA P. gingivalis*	F	TACCCATCGTCGCCTTGGT	[52]
	R	CGGACTAAAACCGCATACACTTG	
*16 S rRNA B. bifidum*	F	TGACCGACCTGCCCCATGCT	[53]
	R	CCCATCCCACGCCGATAGAAT	
*c-MYC*	F	ACCACCAGCAGCGACTCTGA	[54]
	R	TCCAGCAGAAGGTGATCCAGACT	
*lncRNA CCAT2*	F	TGGACTGGAAGTCAAGAGCC	[55]
	R	CCCAGATGCAGAGAACGAGG	
*16 S rRNA universal*	F	GTGSTGCAYGGYTGTCGTCA	[56]
	R	ACGTCRTCCMCACCTTCCTC	
*GAPDH*	F	CCAGGTGGTCTCCTCTGACTT	[57]
	R	GTTGCTGTAGCCAAATTCGTTGT	

**Table 2:** Thermal cycling conditions used for Real-time PCR

gene		Initial Denaturation	Denaturation	Annealing	Extension	Total cycles
*lncRNA CCAT2*	Time	10 min	15 s	30 s	30 s	40
	Temperature	95 °C	95° c	61 °C	72 °C	
*c-MYC*	Time	10 min	15 s	45 s	30 s	
	Temperature	95 °C	95° c	60 °C	72 °C	
*16S rRNA B. bifidum*	Time	5 min	30 s	45 s	30 s	
	Temperature	95 °C	95° c	62 °C	72 °C	
*16S rRNA P. gingivalis*	Time	5 min	30 s	45 s	30 s	
	Temperature	95 °C	94° c	60 °C	72 °C	

**Table 3:** Questionnaire data collected from the cancer and control groups

Variables	Patient group				Control group				*P* value
Current / Former smoker	N	L	M	H	N	L	M	H	*P* = 0.12
	92.85	7.14	0	0	72	20	4	4	
Alcohol consumption	N	L	M	H	N	L	M	H	*P* = 0.82
	89.28	7.14	0	3.57	88	12	0	0	
Prior Antibiotic Exposure (within 30 days)	No		Yes		No		Yes		*P* = 0.26
	61.53		38.46		80		20		
Family history of gastrointestinal cancers	Yes		No		Yes		No		*P* = 0.31
	23.07		76.92		36		64		
History of colonoscopy within the past year	Yes		No		Yes		No		*P* = 0.008
	3.84		96.15		32		68		
History of malignancy in other organs	Yes		No		Yes		No		*P* = 0.64
	11.53		88.64		16		84		
Dental procedures in the past 12 months	Yes		No		Yes		No		*P* = 0.81
	26.92		73.07		24		76		
	52.25		40.74		36		64		

**Table 4:** Prevalence of gastrointestinal disorders in the patient and control groups

Gastrointestinal diseases	Patient group	Control group	*P* value
Peptic ulcer	14.28%	6.66%	*P* = 0.56
Irritable bowel syndrome	0%	12%	*P* = 0.98
Anal abscess	3.5%	0%	*P* = 0.39
*H. pylori* infection	3.5%	4%	*P* = 0.63
Gastroesophageal reflux	3.5%	4%	*P* = 0.63
Colitis	0%	12%	*P* = 0.09
Rectal bleeding	3.5%	0%	*P* = 0.52
Gastritis	0%	4%	*P* = 0.47
Hemorrhoids	0%	6.66%	*P* = 0.47
Colonic polyps	3.5%	24%	*P* = 0.03

**Table 5:** Prevalence of underlying diseases in the patient and control groups

Underlying diseases	Patient group	Control group	*P* value
Hypertension	39.28%	20%	*P* = 0.8
Severe anemia	7.14%	4%	*P* = 0.03
Cardiovascular diseases	10.71%	8%	*P* = 0.55
Obesity	7.14%	0%	*P* = 0.53
Diabetes mellitus	21.42%	4%	*P* = 0.69
Respiratory diseases	3.57%	4%	*P* = 0.93
Hypotension	0%	4%	*P* = 0.47

## Data Availability

No datasets were generated or analysed during the current study.

## Abbreviations

CRC: Colorectal Cancer
lncRNA: Long Non-Coding RNA
CCAT2: Colon Cancer–Associated Transcript 2
c-MYC: Cellular Myelocytomatosis Oncogene
P. gingivalis: Porphyromonas gingivalis
B. bifidum: Bifidobacterium bifidum
qRT-PCR: Quantitative Real-Time Polymerase Chain Reaction
cDNA: Complementary DNA
BMI: Body Mass Index
ROC: Receiver Operating Characteristic
OR: Odds Ratio
CI: Confidence Interval
EMT: Epithelial–Mesenchymal Transition
Wnt: Wingless/Integrated
TCF/LEF: T-Cell Factor / Lymphoid Enhancer Factor
MAPK: Mitogen-Activated Protein Kinase
ERK: Extracellular Signal–Regulated Kinase
NLRP3: NOD-, LRR- and Pyrin Domain–Containing Protein 3
IL-1β: Interleukin-1 beta
iNKT: Invariant Natural Killer T
SCFAs: Short-Chain Fatty Acids
MSI: Microsatellite Instability
ASIRs: Age-Standardized Incidence Rates
GAPDH: Glyceraldehyde-3-Phosphate Dehydrogenase

## References

[1] Matsuda T, Fujimoto A, Igarashi Y. Colorectal Cancer: Epidemiology, Risk Factors, and Public Health Strategies. Digestion. 2025;106(2):91–9. 10.1159/000543921 39938491

[2] Roshandel G, Ghasemi-Kebria F, Malekzadeh R. Colorectal Cancer: Epidemiology, Risk Factors, and Prevention. Cancers. 2024;16(8). 10.3390/cancers16081530 PMC11049480 38672612

[3] Zhao H, Ming T, Tang S, Ren S, Yang H, Liu M, et al. Wnt signaling in colorectal cancer: pathogenic role and therapeutic target. Mol Cancer. 2022;21(1):144. 10.1186/s12943-022-01616-7 PMC9281132 35836256

[4] Dang CV. c-Myc target genes involved in cell growth, apoptosis, and metabolism. Mol Cell Biol. 1999;19(1):1–11. 10.1128/mcb.19.1.1 PMC83860 9858526

[5] Wallbillich NJ, Lu H. Role of c-Myc in lung cancer: Progress, challenges, and prospects. Chin Med J pulmonary Crit care Med. 2023;1(03):129–38. 10.1016/j.pccm.2023.07.001 PMC10621893 37920609

[6] Dang CV, O’Donnell KA, Zeller KI, Nguyen T, Osthus RC, Li F. The c-Myc target gene network. Sem Cancer Biol. 2006;16(4):253–64. 10.1016/j.semcancer.2006.07.014 16904903

[7] Feng XH, Liang YY, Liang M, Zhai W, Lin X. Direct interaction of c-Myc with Smad2 and Smad3 to inhibit TGF-beta-mediated induction of the CDK inhibitor p15(Ink4B). Mol Cell. 2002;9(1):133–43. 10.1016/s1097-2765(01)00430-0 11804592

[8] Erisman MD, Rothberg PG, Diehl RE, Morse CC, Spandorfer JM, Astrin SM. Deregulation of c-myc gene expression in human colon carcinoma is not accompanied by amplification or rearrangement of the gene. Mol Cell Biol. 1985;5(8):1969–76. 10.1128/mcb.5.8.1969-1976.1985 PMC366914 3837853

[9] Rowley S, Newbold KM, Gearty J, Keighley MR, Donovan IA, Neoptolemos JP. Comparison of deoxyribonucleic acid ploidy and nuclear expressed p62 c-myc oncogene in the prognosis of colorectal cancer. World J Surg. 1990;14(4):545–50. discussion 51. 10.1007/BF01658688 2200214

[10] Bhatavdekar JM, Patel DD, Ghosh N, Chikhlikar PR, Trivedi TI, Suthar TP, et al. Coexpression of Bcl-2, c-Myc, and p53 oncoproteins as prognostic discriminants in patients with colorectal carcinoma. Dis Colon Rectum. 1997;40(7):785–90. 10.1007/BF02055433 9221853

[11] Kakisako K, Miyahara M, Uchino S, Adachi Y, Kitano S. Prognostic significance of c-myc mRNA expression assessed by semi-quantitative RT-PCR in patients with colorectal cancer. Oncol Rep. 1998;5(2):441–5. 10.3892/or.5.2.441 9468576

[12] Fang Y, Shen ZY, Zhan YZ, Feng XC, Chen KL, Li YS, et al. CD36 inhibits β-catenin/c-myc-mediated glycolysis through ubiquitination of GPC4 to repress colorectal tumorigenesis. Nat Commun. 2019;10(1):3981. 10.1038/s41467-019-11662-3 PMC6726635 31484922

[13] Kim J, Lee HK. Potential Role of the Gut Microbiome. In Colorectal Cancer Progression. 2022;Volume 12–2021. 10.3389/fimmu.2021.807648 PMC8777015 35069592

[14] Ahmad Kendong SM, Raja Ali RA, Nawawi KNM, Ahmad HF, Mokhtar NM. Gut dysbiosis and intestinal barrier dysfunction: potential explanation for early-onset colorectal cancer. Front Cell Infect Microbiol. 2021;11:744606. 10.3389/fcimb.2021.744606. PMID: 34966694; PMCID: PMC8710575. PMC8710575 34966694

[15] Galasso L, Termite F, Mignini I, Esposto G, Borriello R, Vitale F, et al. Unraveling the role of Fusobacterium nucleatum in colorectal cancer: molecular mechanisms and pathogenic insights. Cancers (Basel). 2025;17(3):368. 10.3390/cancers17030368. PMID: 39941737; PMCID: PMC11816155. PMC11816155 39941737

[16] Rubinstein MR, Baik JE, Lagana SM, Han RP, Raab WJ, Sahoo D, et al. *Fusobacterium nucleatum* promotes colorectal cancer by inducing Wnt/β‐catenin modulator Annexin A1. EMBO reports. 2019;20(4):e47638. 10.15252/embr.201847638 PMC6446206 30833345

[17] Sun CH, Li BB, Wang B, Zhao J, Zhang XY, Li TT, et al. The role of Fusobacterium nucleatum in colorectal cancer: from carcinogenesis to clinical management. Chronic Dis translational Med. 2019;5(3):178–87. 10.1016/j.cdtm.2019.09.001 PMC6926109 31891129

[18] Ranjbar M, Salehi R, Haghjooy Javanmard S, Rafiee L, Faraji H, jafarpor S, et al. The dysbiosis signature of Fusobacterium nucleatum in colorectal cancer-cause or consequences? A systematic review. Cancer Cell Int. 2021;21(1):194. 10.1186/s12935-021-01886-z PMC8025348 33823861

[19] Wang X, Jia Y, Wen L, Mu W, Wu X, Liu T, et al. Porphyromonas gingivalis promotes colorectal carcinoma by activating the hematopoietic NLRP3 inflammasome. Cancer Res. 2021;81(10):2745–59. 10.1158/0008-5472.CAN-20-3827. Epub 2021 Mar 18. Erratum in: Cancer Res. 2022;82(11):2196. PMID: 34003774. 34003774

[20] Maekawa T, Krauss JL, Abe T, Jotwani R, Triantafilou M, Triantafilou K, et al. Porphyromonas gingivalis manipulates complement and TLR signaling to uncouple bacterial clearance from inflammation and promote dysbiosis. Cell Host Microbe. 2014;15(6):768–78. 10.1016/j.chom.2014.05.012 PMC4071223 24922578

[21] Mu W, Jia Y, Chen X, Li H, Wang Z, Cheng B. Intracellular Porphyromonas gingivalis Promotes the Proliferation of Colorectal Cancer Cells via the MAPK/ERK Signaling Pathway. Front Cell Infect Microbiol. 2020;10:584798. 10.3389/fcimb.2020.584798 PMC7785964 33425779

[22] Díaz-Basabe A, Lattanzi G, Perillo F, Amoroso C, Baeri A, Farini A, et al. Porphyromonas gingivalis fuels colorectal cancer through CHI3L1-mediated iNKT cell-driven immune evasion. Gut Microbes. 2024;16(1):2388801. 10.1080/19490976.2024.2388801 PMC11321422 39132842

[23] Chandrasekaran P, Weiskirchen S, Weiskirchen R. Effects of Probiotics on Gut Microbiota. Overv. 2024;25(11):6022. 10.3390/ijms25116022 PMC11172883 38892208

[24] Hemarajata P, Versalovic J. Effects of probiotics on gut microbiota: mechanisms of intestinal immunomodulation and neuromodulation. Therapeutic Adv Gastroenterol. 2013;6(1):39–51. 10.1177/1756283X12459294 PMC3539293 23320049

[25] Parada Venegas D, De la Fuente MK, Landskron G, González MJ, Quera R, Dijkstra G, et al. Short chain fatty acids (SCFAs)-mediated gut epithelial and immune regulation and its relevance for inflammatory bowel diseases. Front Immunol. 2019;10:277. 10.3389/fimmu.2019.00277. PMID: 30915065; PMCID: PMC6421268. PMC6421268 30915065

[26] Zhang Z, Zhang H, Chen T, Shi L, Wang D, Tang D. Regulatory role of short-chain fatty acids in inflammatory bowel disease. Cell Communication Signal. 2022;20(1):64. 10.1186/s12964-022-00869-5 PMC9097439 35546404

[27] Mirzaei R, Afaghi A, Babakhani S, Sohrabi MR, Hosseini-Fard SR, Babolhavaeji K, et al. Role of microbiota-derived short-chain fatty acids in cancer development and prevention. Biomed Pharmacother. 2021;139:111619. 10.1016/j.biopha.2021.111619 33906079

[28] Zhao J, Liao Y, Wei C, Ma Y, Wang F, Chen Y, et al. Potential Ability of Probiotics in the Prevention and Treatment of Colorectal Cancer. Clin Med Insights Oncol. 2023;17:11795549231188225. 10.1177/11795549231188225 PMC10437046 37601319

[29] Yao S, Zhao Z, Wang W, Liu X. Bifidobacterium Longum: Prot against Inflamm Bowel Disease. 2021;2021(1):8030297. 10.1155/2021/8030297 PMC8324359 34337079

[30] Gavzy SJ, Kensiski A, Lee ZL, Mongodin EF, Ma B, Bromberg JS. Bifidobacterium mechanisms of immune modulation and tolerance. Gut Microbes. 2023;15(2):2291164. 10.1080/19490976.2023.2291164 PMC10730214 38055306

[31] Faghfoori Z, Faghfoori MH, Saber A, Izadi A, Yari Khosroushahi A. Anticancer effects of bifidobacteria on colon cancer cell lines. Cancer Cell Int. 2021;21(1):258. 10.1186/s12935-021-01971-3 PMC8114702 33980239

[32] Benito I, Encío IJ, Milagro FI, Alfaro M, Martínez-Peñuela A, Barajas M et al. Microencapsulated Bifidobacterium bifidum and Lactobacillus gasseri in Combination with Quercetin Inhibit Colorectal Cancer Development in Apc(Min/+) Mice. Int J Mol Sci. 2021;22(9). 10.3390/ijms22094906 PMC8124226 34063173

[33] Yin J, Chen L, Lin Y, Qiu J, Liu F, Wang Y, et al. Bifidobacterium bifidum reduces oxidative stress and alters gut flora to mitigate acute liver injury caused by N-acetyl-p-aminophenol. BMC Microbiol. 2025;25(1):87. 10.1186/s12866-025-03775-1 PMC11853282 40000948

[34] Ramesh A, Srinivasan D, Subbarayan R, Chauhan A, Krishnamoorthy L, Kumar J, et al. Enhancing colorectal cancer treatment: the role of Bifidobacterium in modulating gut immunity and mitigating capecitabine-induced toxicity. Mol Nutr Food Res. 2025;69(9):e70023. 10.1002/mnfr.70023. Epub 2025 Mar 20. PMID: 40109200. 40109200

[35] Kong Y, Bai H, Deng F, Zhao Y, Li Q, Chang L. Application of bifidobacterium in tumor therapy. Front Oncol. 2025;15:1551924. 10.3389/fonc.2025.1551924. PMID: 40469191; PMCID: PMC12134754. PMC12134754 40469191

[36] Amonkar R, Uy AA, Ramirez P, Patel H, Jeong JJ, Shoyele NO et al. Engineered bifidobacterium strains colonization at tumor sites: a novel approach to the delivery of cancer treatments. Cancers. 2025;17(15). 10.3390/cancers17152487 PMC12346535 40805186

[37] He Q, Long J, Yin Y, Li Y, Lei X, Li Z, et al. Emerging Roles of lncRNAs in the Formation and Progression of Colorectal Cancer. Front Oncol. 2019;9:1542. 10.3389/fonc.2019.01542 PMC6978842 32010629

[38] Siddiqui H, Al-Ghafari A, Choudhry H, Al Doghaither H. Roles of long non-coding RNAs in colorectal cancer tumorigenesis: A Review. Mol Clin Oncol. 2019;11(2):167–72. 10.3892/mco.2019.1872 PMC6589935 31281651

[39] Fathollahzadeh M, Vanan AG, Mohammadmoradi N, Ghorbaninezhad F, Eini P, Tofighi Zavareh MA, et al. The *CCAT2* enigma: pioneering insights into colorectal cancer pathophysiology and therapeutic innovation. Cancer Cell Int. 2025;26(1):27. 10.1186/s12935-025-04086-1 PMC12821247 41310609

[40] Ling H, Spizzo R, Atlasi Y, Nicoloso M, Shimizu M, Redis RS, et al. *CCAT2*, a novel noncoding RNA mapping to 8q24, underlies metastatic progression and chromosomal instability in colon cancer. Genome Res. 2013;23(9):1446–61. 10.1101/gr.152942.112 PMC3759721 23796952

[41] Wang L, Duan W, Yan S, Xie Y, Wang C. Circulating long non-coding RNA colon cancer-associated transcript 2 protected by exosome as a potential biomarker for colorectal cancer. Biomed Pharmacother. 2019;113:108758. 10.1016/j.biopha.2019.108758. Epub 2019 Mar 14. PMID: 30877883. 30877883

[42] Zhang X, Qi J, Dong C, Zhang L, Chen J, Liu J et al. Intratumoral microbiota in colorectal cancer: roles, therapeutic potential, and challenges. J Adv Res. 2025. 10.1016/j.jare.2025.11.067 PMC13539154 41338385

[43] Morsli DS, Tbahriti HF, Rahli F, Mahammi FZ, Nagdalian A, Hemeg HA, et al. Probiotics in colorectal cancer prevention and therapy: mechanisms, benefits, and challenges. Discover Oncol. 2025;16(1):406. 10.1007/s12672-025-01996-4 PMC11947384 40140210

[44] Amitay EL, Carr PR, Jansen L, Roth W, Alwers E, Herpel E, et al. Smoking, alcohol consumption and colorectal cancer risk by molecular pathological subtypes and pathways. Br J Cancer. 2020;122(11):1604–10. 10.1038/s41416-020-0803-0 PMC7250912 32225169

[45] Pala V, Sieri S, Berrino F, Vineis P, Sacerdote C, Palli D, et al. Yogurt consumption and risk of colorectal cancer in the Italian European prospective investigation into cancer and nutrition cohort. Int J Cancer. 2011;129(11):2712–9. 10.1002/ijc.26193. Epub 2011 Aug 5. PMID: 21607947. 21607947

[46] Liu Z-H, Huang M-J, Zhang X-W, Wang L, Huang N-Q, Peng H, et al. The effects of perioperative probiotic treatment on serum zonulin concentration and subsequent postoperative infectious complications after colorectal cancer surgery: a double-center and double-blind randomized clinical trial1 2 3 4. Am J Clin Nutr. 2013;97(1):117–26. 10.3945/ajcn.112.040949 23235200

[47] Kerdreux M, Edin S, Löwenmark T, Bronnec V, Löfgren-Burström A, Zingmark C, et al. Porphyromonas gingivalis in Colorectal Cancer and its Association to Patient Prognosis. J Cancer. 2023;14(9):1479–85. 10.7150/jca.83395 PMC10266249 37325051

[48] Heydari Z, Rahaie M, Alizadeh AM, Agah S, Khalighfard S, Bahmani S. Effects of Lactobacillus acidophilus and Bifidobacterium bifidum Probiotics on the Expression of MicroRNAs 135b, 26b, 18a and 155, and Their Involving Genes in Mice Colon Cancer. Probiotics Antimicrob proteins. 2019;11(4):1155–62. 10.1007/s12602-018-9478-8 30311185

[49] Esfandiari F, Bakhshi B, Shahbazi T, Derakhshan-nezhad E, Bahroudi M, Minaeeian S, et al. Significant difference in gut microbiota Bifidobacterium species but not Lactobacillus species in colorectal cancer patients in comparison with healthy volunteers using quantitative real-time PCR. PLoS ONE. 2024;19(11):e0294053. 10.1371/journal.pone.0294053 PMC11602092 39602380

[50] Maleki Moaf Z, Salehzadeh A, Zamanzadeh Z. Investigation of the expression of LncRNA/*CCAT2* gene in patients with colorectal cancer. J Epigenetics. 2021;2(2):39–44.

[51] Gao P, Sun D, Guo H, Wu Z, Chen J. LncRNA *CCAT2* promotes proliferation and suppresses apoptosis of colorectal cancer cells. J BUON: official J Balkan Union Oncol. 2020;25(4):1840–6. 33099922

[52] Kawada M, Yoshida A, Suzuki N, Nakano Y, Saito T, Oho T, et al. Prevalence of Porphyromonas gingivalis in relation to periodontal status assessed by real-time PCR. Oral Microbiol Immunol. 2004;19(5):289–92. 10.1111/j.1399-302X.2004.00154.x 15327639

[53] Rinne MM, Gueimonde M, Kalliomäki M, Hoppu U, Salminen SJ, Isolauri E. Similar bifidogenic effects of prebiotic-supplemented partially hydrolyzed infant formula and breastfeeding on infant gut microbiota. FEMS Immunol Med Microbiol. 2005;43(1):59–65. 10.1016/j.femsim.2004.07.005 15607637

[54] Hawksworth D, Ravindranath L, Chen Y, Furusato B, Sesterhenn IA, McLeod DG, et al. Overexpression of C-MYC oncogene in prostate cancer predicts biochemical recurrence. Prostate Cancer Prostatic Dis. 2010;13(4):311–5. 10.1038/pcan.2010.31 20820186

[55] Gao H, Wang X, Ma H, Lin S, Zhang D, Wu W, et al. LncRNA *CCAT2*, involving miR-34a/TGF-β1/Smad4 signaling, regulate hepatic stellate cells proliferation. Sci Rep. 2022;12(1):21199. 10.1038/s41598-022-25738-6 PMC9732356 36482069

[56] Forster SC, Clare S, Beresford-Jones BS, Harcourt K, Notley G, Stares MD, et al. Identification of gut microbial species linked with disease variability in a widely used mouse model of colitis. Nat Microbiol. 2022;7(4):590–9. 10.1038/s41564-022-01094-z PMC8975739 35365791

[57] Dong Y, Wang J, Sheng Z, Li G, Ma H, Wang X, et al. Downregulation of EphA1 in colorectal carcinomas correlates with invasion and metastasis. Mod pathology: official J United States Can Acad Pathol Inc. 2009;22(1):151–60. 10.1038/modpathol.2008.188 19011600

